# Parthenocarpic tomato mutants, *iaa9-3* and *iaa9-5*, show plant adaptability and fruiting ability under heat-stress conditions

**DOI:** 10.3389/fpls.2023.1090774

**Published:** 2023-03-01

**Authors:** Syariful Mubarok, Nurul Jadid, Ani Widiastuti, Deden Derajat Matra, Rahmat Budiarto, Fitrianti Widya Lestari, Anne Nuraini, Erni Suminar, Bayu Pradana Nur Rahmat, Hiroshi Ezura

**Affiliations:** ^1^ Department of Agronomy, Faculty of Agriculture, Universitas Padjadjaran, Sumedang, Indonesia; ^2^ Department of Biology, Institut Teknologi Sepuluh Nopember, Surabaya, Indonesia; ^3^ Department of Plant Protection, Faculty of Agriculture, Universitas Gadjah Mada, Yogyakarta, Indonesia; ^4^ Department of Agronomy and Horticulture, Faculty of Agriculture, IPB University, Bogor, Indonesia; ^5^ Plantation Seed Supervision and Certification Center, Bandung, Indonesia; ^6^ Master Graduate Program of Agronomy, Faculty of Agriculture, Universitas Padjadjaran, Sumedang, Indonesia; ^7^ Faculty of Life and Environmental Sciences, University of Tsukuba, Tsukuba, Japan; ^8^ Tsukuba Plant Innovation Research Center, University of Tsukuba, Tsukuba, Japan

**Keywords:** tomato, parthenocarpic, auxin, heat stress, mutant

## Abstract

Fruit set is one of the main problems that arise in tomato plants under heat-stress conditions, which disrupt pollen development, resulting in decreased pollen fertility. Parthenocarpic tomatoes can be used to increase plant productivity during failure of the fertilisation process under heat-stress conditions. The aim of this study were to identify the plant adaptability and fruiting capability of ?*iaa9-3* and *iaa9-5* tomato mutants under heat-stress conditions. The *iaa9-3* and *iaa9-5* and wild-type Micro-Tom (WT-MT) plants were cultivated under two temperature conditions: normal and heat-stress conditions during plant growth. The results showed that under the heat-stress condition, *iaa9-3* and *iaa9-5* showed delayed flowering time, increased number of flowers, and increased fruit set and produced normal-sized fruit. However, WT-MT cannot produce fruits under heat stress. The mutants can grow under heat-stress conditions, as indicated by the lower electrolyte leakage and H_2_O_2_ concentration and higher antioxidant activities compared with WT-MT under heat-stress conditions. These results suggest that *iaa9-3* and *iaa9-5* can be valuable genetic resources for the development of tomatoes in high-temperature environmental conditions.

## 1 Introduction

Tomato (*Solanum lycopersicum*) is an essential Solanaceae horticultural commodity in the world (Anas et al., 2022). Tomatoes are generally used as a source of nutrients and antioxidants, such as vitamins A, C and B, calcium, potassium, magnesium, sodium, iron, phenolic antioxidants, flavonoids, lycopene and ascorbic acid (Oboulbiga et al., 2017; Mubarok et al., 2019a; Mubarok et al., 2023). Tomato production is affected by several factors, such as genetics, nutrition and cultivation methods and environmental factors, including temperature.

Temperature is a very important environmental factor for the growth and development of tomato plants. Heat stress is an external factor that often becomes an obstacle to tomato cultivation. In general, heat stress occurs when temperatures are 10 °C–15 °C higher than optimum cultivation temperatures; for tomato growth, the optimum temperature is 26 °C and heat stress starts to occur when air temperature exceeds 32 °C (Sato et al., 2000; Pham et al., 2020). Heat stress is one of the principal abiotic stresses in tropical countries that limit plant growth and development, affecting production. Failure in pollen development and increased pollen sterility are the main problems in tomato production under heat stress, resulting in a low fruit set (Ezura et al., 2019). The Intergovernmental Panel on Climatic Change reported that in 2014, each country’s average world temperature increased by 0.3°C due to global warming and is predicted to increase by 3°C from the usual temperature in 2100. Increased temperature due to global warming has become a severe problem in the agricultural sector. Thus, new tomato cultivars suitable for high-temperature conditions must be developed.

Environmental temperatures above 35 °C in tomato cultivation damage plant growth and development, including seed germination, vegetative and reproductive growth, fruit setting and photosynthetic activity (Ariizumi et al., 2013; Rahmat et al., 2023). The severity of heat stress depends on the duration and time of heat-stress exposure. Ariizumi et al. (2013) reported that ambient temperature 4 degrees above optimal temperature will increase tomato flower abortion. Flower abortion occurred due to the failure of pollination and fertilisation, and flower abortion reduces fruit set initiation. Generation of parthenocarpic tomatoes can be an alternative option to resolving the problem in fruit set initiation under heat-stress conditions, which causes reduced fruit formation due to pollen sterility.

Auxin (AUX) triggers fruit set in tomatoes by activating cell division in the pericarp (i.e. phase II); therefore, induction of parthenocarpic fruits can be artificially attained by the application of exogenous AUX in tomato flowers or flower buds (Ariizumi et al., 2013). By mutation methods, two parthenocarpic tomatoes, namely, *iaa9-3* and *iaa9-5*, have been generated from the Micro-Tom (MT) library. Compared with the wild type, *iaa9-3* and *iaa9-5* produced higher rates of parthenocarpic fruit production, with values reaching up to 70.0% ± 4.7% and 63.3% ± 5.4%, respectively, from an emasculated flower. Meanwhile, in the wild-type MT (WT-MT), almost none of the emasculated WT-MT flowers exhibited fruit formation (Saito et al., 2011).

*iaa9-3* and *iaa9-5* have a mutation in *IAA9*, which is a member of the AUX/indole-3-acetic acid (IAA) gene family and acts as a transcriptional repressor of the signalling pathway of the plant hormone AUX (Guilfoyle and Hagen, 2007). Different locations of mutations occur in *iaa9-3* and *iaa9-5*. In *iaa9-3*, a single-DNA substitution causing a T to A substitution was found at the 237th nucleotide, whereas in *iaa9-5*, a 32 bp deletion was located at the 133rd nucleotide position. These mutations by DNA substitution or deletion in these mutants result in the modification of the functional activity of *IAA9*, which triggers fruit development prior to pollination or parthenocarpy (Saito et al., 2011).

Parthenocarpic mutants, such as *iaa9-3* and *iaa9-5*, can produce fruits under heat-stress conditions and help maintain stable tomato production in the future. Those two mutants also can growth under drought stress (Suminar et al., 2022). However, the ability of such mutants for plant growth and development, especially in the fruit set under heat-stress conditions, should be carefully evaluated. In this study, we evaluated the fruit set ability of two mutants under tropical heat-stress conditions.

## 2 Materials and methods

### 2.1 Plant materials and cultivation condition

The *iaa9-3* (TOMPJE2811) and *iaa9-5* (TOMJPG0114-1) mutants were isolated and characterised from a mutant population based on MT cultivars (Saito et al., 2011). These mutants and WT-MT as control were cultivated in the greenhouse of the experimental field of the Laboratory of Horticulture, Faculty of Agriculture, Universitas Padjadjaran, with four biological replications; each replication consisted of six individual plants for vegetative analysis. The *iaa9-3* and *iaa9-5* mutants and WT-MT seeds were sown in plug trays, in accordance with the methods described by Rahmat et al. (2023). After the seedling produced 4–5 leaves, they were transplanted into a 12 cm-diameter pot that was filled with a mixture of soil and commercial coir coco peat (1:1/v:v) and cultivated in two greenhouse conditions, i.e. heat-stress (40 °C/45 °C) and control (30 °C/35 °C) conditions. The heat stress condition was treated for the whole lifespan of the plant, starting from transplanting until the fruit harvest. During the cultivation periods, the plants were fertigated with a commercial nutrient solution with an electrical conductivity (EC) level of 2.0–2.5 dS m^−1^.

### 2.2 Agronomic-related yield evaluation of iaa9-3 and iaa9-5 mutants

The agronomic features related to plant growth and yield were evaluated. For plant growth, plant canopy diameter that measured as the method described by Iizuka et al. (2017) and the number of shoots were evaluated at 8 weeks after sowing. For plant yield, several characteristics were evaluated; these characteristics included flowering time that was the number of days from sowing until 1^st^ flowering from each plant (Mubarok et al., 2019c), the number flowers per plant, the number of fruits per plant, the rate of fruit set, fruit weight, length and diameter and the number of seeds. The fruit weight per plant was calculated by dividing the total fruit weight by the total fruit number. The total number of flowers and fruits and the rate of fruit set were counted similarly to those in the study by Pham et al. (2020) at 115 days after sowing. Meanwhile, the rate of fruit set was calculated by dividing the total number of fruits by the total number of flowers. The fruit weight, length and diameter and the number of seeds per fruit were obtained from five individual fruits of each plant.

### 2.3 Pollen viability analysis

Pollen viability testing was carried out using 2,3,5-triphenyl tetrazolium chloride (TTC) in accordance with the method used by Ezura et al. (2019). TTC staining is an indicator of cellular survival based on the mitochondrial reduction reaction, where the viable pollen will turn red, and the non-viable pollen will be colourless. Pollens were obtained from five individual flowers, soaked in 1% TTC +50% sucrose and shaken for dispersal in the solution. Afterwards, the TTC solution containing the pollens was left in a dark room at 38°C for 3 h for pollen colouration. Then, 2 µg supernatant was collected and placed in the cell counter. The pollens were then observed under a microscope. The criterion for viable pollens was the absorption of red colour from the TTC solution per 1 mm^2^ cell counter.

### 2.4 H_2_O_2_ concentration analysis

H_2_O_2_ concentration was measured following the method used by the Association of Official Analytical Chemists (AOAC) with modifications (Association of Official Analytical Chemists, 1990). A total of 5 g leaf sample was mixed with 5 mL Aqua Bidest using mortar and pestle in an ice bath. Then, 5 mL ground leaf sample mixture was combined with 25 mL sulfuric acid. The mixture was titrated with 0.1 N potassium permanganate. Each 1 mL of potassium permanganate used in titration corresponded to 1.07 mg H_2_O_2_. Titration was stopped when the sample mixture changed colour. H_2_O_2_ was calculated using the following formula:


$$H_{2}O_{2\;}=\frac{V\times 0.1\;\left(KMnO_{4}\right)\times \;1.701}{W\times 0.01\;\left(H_{2}SO_{4}\right)}\;\times 100\%$$

V = Titrate used in titration (mL)

W = Weight of the leaf sample (g)

The H_2_O_2_ values were then converted from percentage to µmol/g of fresh weight (FW).

### 2.5 Antioxidant activity analysis

Antioxidant activity was analysed following the method used by Sami and Rahimah (2016). A total of 3 g leaf sample was macerated using methanol (99.8%) for 24 h. After 24 h, the mixture was filtrated using filter paper, and the filtrate was thickened utilising a rotary evaporator. The leaf extract solution (1000 ppm) was created by combining 50 mg thickened leaf extract with 50 mL methanol (99.8%). Next, 0.5 mL 0.4 mM 2,2- diphenyl-2-picrylhydrazyl (DPPH) solution was added to a test tube containing 500 µL leaf extract solution (1000 ppm). The test tubes containing the mixture were then incubated in the dark for 30 min at room temperature. After incubation, each sample absorbance was observed using a spectrophotometer (Orion™ AquaMate 8000, Thermo Fisher Scientific, UK) at 516 nm (Mubarok et al., 2019b). A blank solution containing methanol and DPPH only was also prepared. Sample antioxidant activity was measured using the following equation:


$$Antioxidant\;activity\;\left(\%\right)=\frac{Blank\;absorbance-Sample\;absorbance}{Blank\;absorbance}\times 100\%$$

### 2.6 Statistical data analysis

The normalities of data distributions were tested using the Kolmogorov–Smirnov test. For statistical data analysis, data were subjected to a two-factor analysis of variance followed by Duncan’s multiple range test at p< 0.05 for comparisons among the investigated data. The data are represented as the mean values ± standard error (SE) of four replicates. All statistical analyses were performed using SPSS 20.0 statistical software.

## 3 Results

### 3.1 iaa9-3 and iaa9-5 exhibited a reduction in vegetative plant growth compared with WT-MT

**Figure 1:**
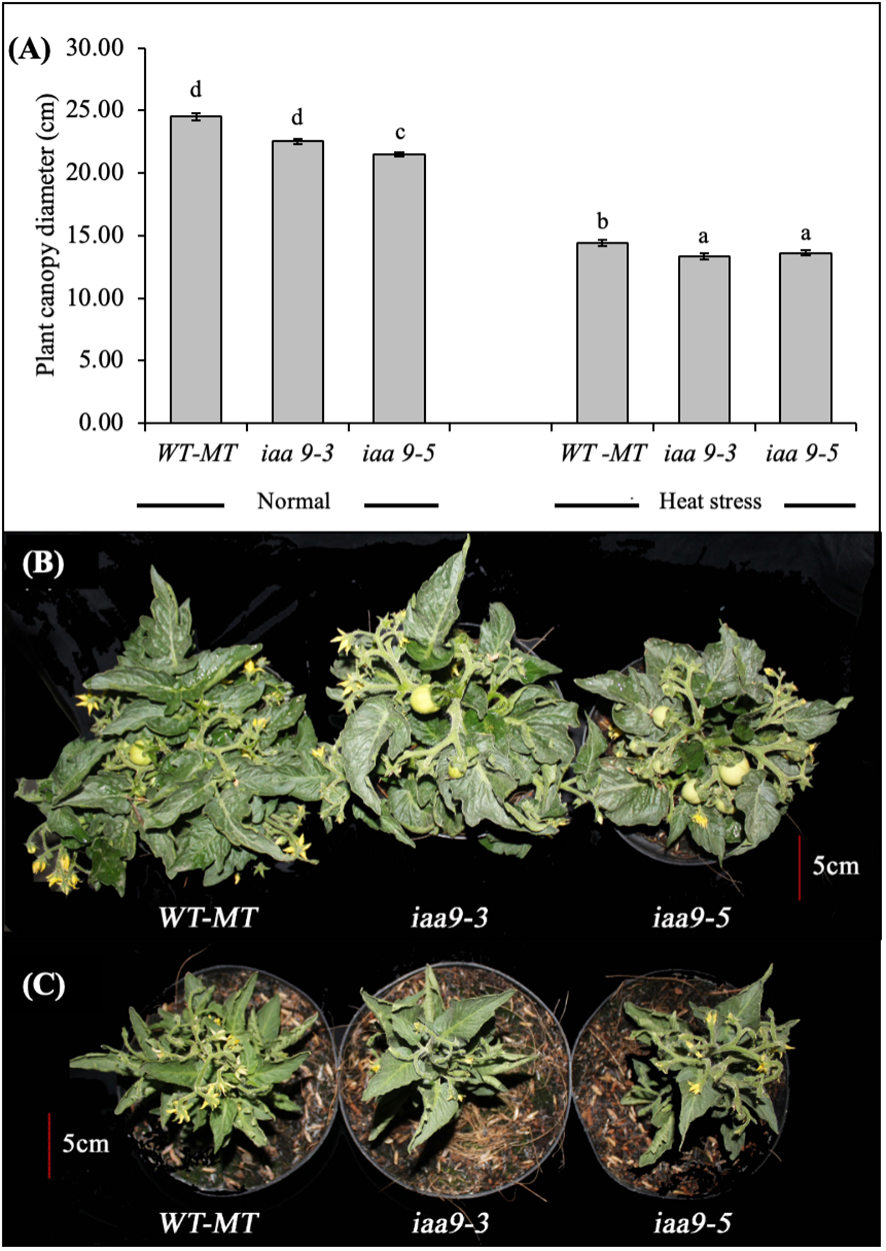
**(A)** The average value of the plant canopy diameter of WT-MT, *iaa9-3*, and *iaa9-5* under two temperature conditions. The plant canopy of WT-MT, *iaa9-3* and *iaa9-5* under **(B)** normal temperature and **(C)** heat stress condition. The average value ± standard error (SE) (n = 4) followed by the same lowercase letters is not significantly different based on Duncan’s Multiple Range Test at 5%.

The response of plant vegetative growth can be observed from the plant appearance, which was characterised by the plant canopy diameter and the number of shoots. Based on statistical data analysis, the plant canopy diameter of WT-MT, *iaa9-3* and *iaa9-5* mutants were significantly different in either normal temperature or heat-stress conditions (**Figures 1A–C**, respectively). High-temperature/heat-stress condition greatly influenced the size of the plant canopy diameter. Under heat-stress conditions, the plant canopy diameter decreased significantly in all plant genotypes compared with plants under normal conditions. The highest reduction in the plant canopy diameter was observed in WT-MT, and it decreased by approximately 41.3% compared with that under normal condition. The plant canopy diameters of *iaa9-3* and *iaa9-5* decreased by 40.6% and 36.7%, respectively (**Figures 1A, C**).

At both temperatures tested, the plant canopy diameter of WT-MT was larger than those of *iaa9-3* and *iaa9-5*. As shown in **Figure 1A**, the plant canopy diameter of WT-MT under normal conditions showed the highest diameter, but it was not significantly different from that of *iaa9-3*. Meanwhile, *iaa9-5* had significantly lower values compared with WT-MT and *iaa9-3* (**Figure 1A**). The large plant canopy diameter of WT-MT can also be monitored under heat-stress condition, where the plant canopy diameters of *iaa9-3* and *iaa9-5* were significantly lower than that of WT-MT (**Figure 1A**).

**Figure 2:**
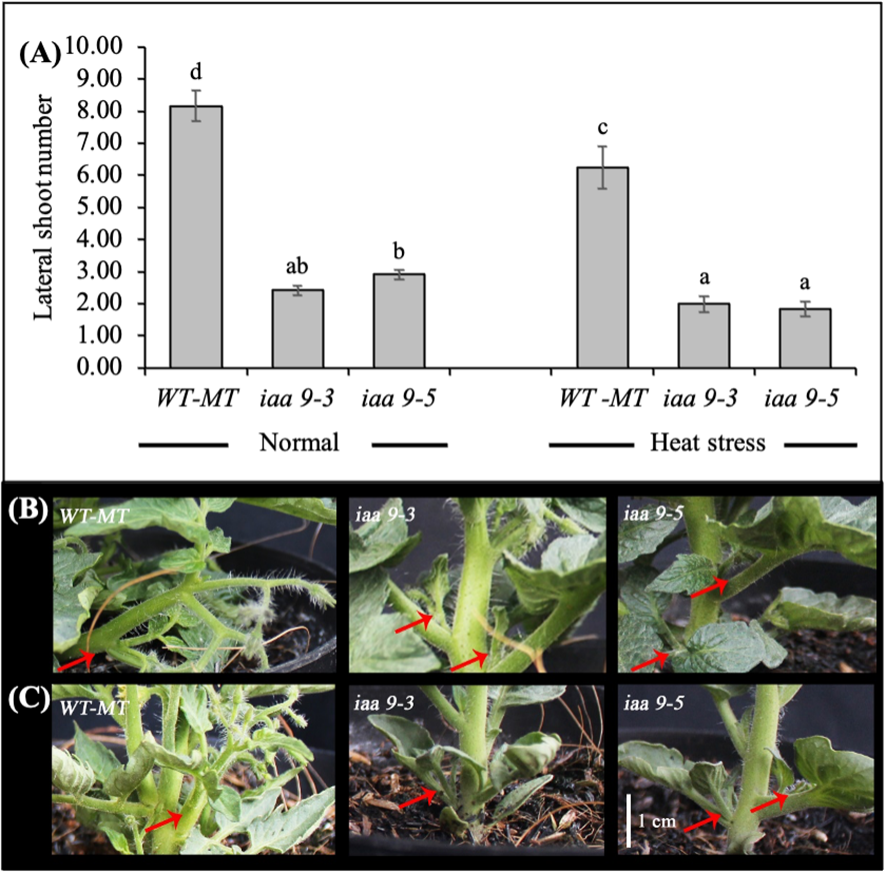
**(A)** The average value of lateral shoot number of WT-MT, *iaa9-3* and *iaa9-5* under two temperature conditions. The lateral shoot number of WT-MT, *iaa9-3* and *iaa9-5* under **(B)** normal temperature and **(C)** heat stress condition. The average value ± standard error (SE) (n = 4) followed by the same lowercase letters is not significantly different based on Duncan’s Multiple Range Test at 5%.

Many lateral shoots caused the large plant canopy diameter of WT-MT. Statistical data analysis showed a significant difference in the number of lateral shoots between WT-MT and *iaa9-3* and *iaa9-5* under normal and heat-stress conditions. The heat-stress condition significantly inhibited the growth of new lateral shoots in all investigated plant genotypes, which resulted in a low number of lateral shoots under heat-stress conditions. However, no further reduction in lateral shoots was observed in *iaa9-3* under heat stress (**Figure 2B**). The decrease in the number of lateral shoots under heat-stress condition was 23.50%, 17.35% and 38.36% lower compared with those under normal conditions for WT-MT, *iaa9-3* and *iaa9-5*, respectively. Under normal and heat-stress conditions, the mutation in the *IAA9* gene significantly reduced the number of lateral shoots in *iaa9-3* and *iaa9-5*. In *iaa9-3*, the number of lateral shoots was 5.75 and 4.25 fewer compared with that in WT-MT under normal and heat-stress conditions, respectively. *Iaa9-5* showed 5.25 and 4.45 fewer lateral shoots compared with WT-MT under normal and heat-stress conditions, respectively (**Figures 2A–C**).

### 3.2 Mutant genotypes iaa9-3 and iaa9-5 experienced delayed flowering under the heat-stress condition

**Figure 3:**
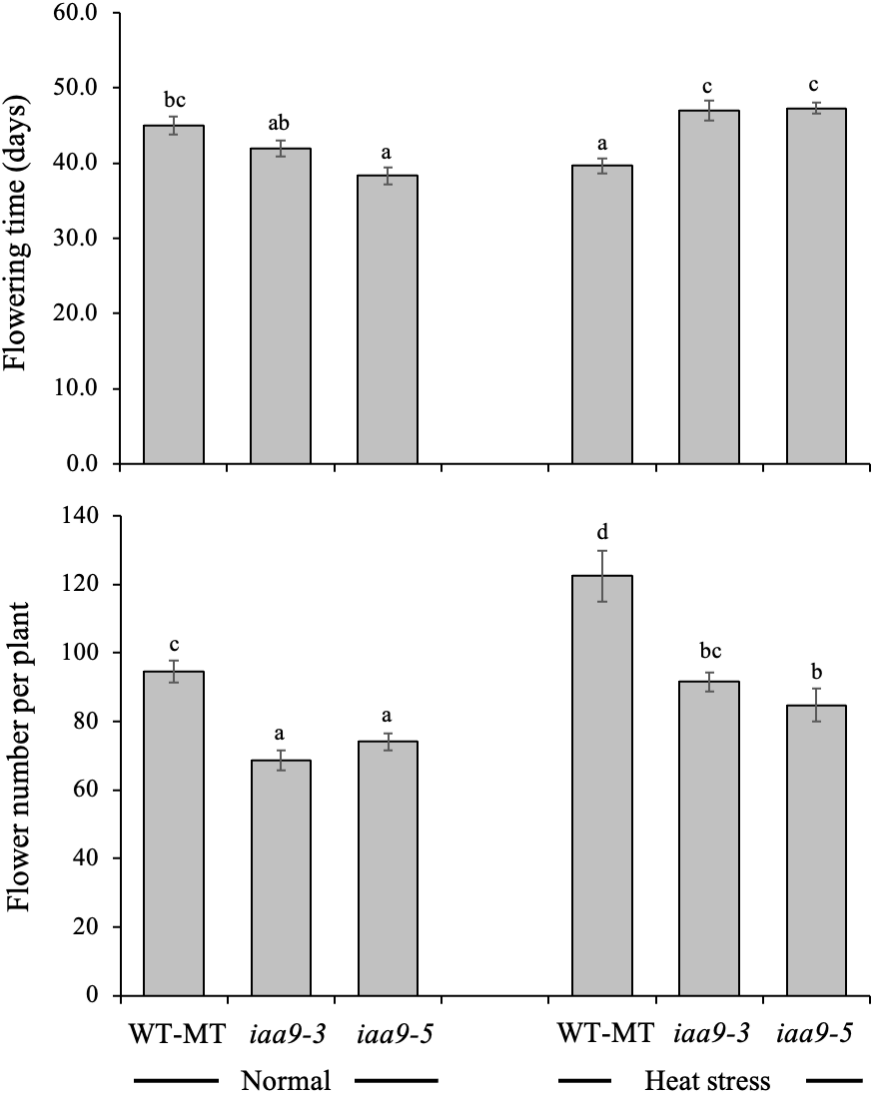
Flowering time and flower number per plant of WT-MT, *iaa9-3* and *iaa9-5* under two temperature conditions, i.e., normal temperature and heat stress condition. The average value ± standard error (SE) (n = 4) followed by the same lowercase letters is not significantly different based on Duncan’s Multiple Range Test at 5%.

Flowering is strongly influenced by internal and external factors. The time of flowering is one of the several indicators of a plant entering the generative phase. The results showed that the flowering time of the three observed tomato genotypes, namely, *iaa9-3*, *iaa9-5* and WT-MT, differed depending on environmental conditions. Under the normal-temperature condition, the mutation in *iaa9-3* and *iaa9-5* significantly accelerated flowering time by 3 and 6.7 days earlier than WT-MT, respectively (**Figure 3**). This result was different from that observed under heat-stress condition. Accelerated flowering time was observed in WT-MT, but *iaa9-3* and *iaa9-5* experienced a delay in flowering time. The mutants behaved as WT under normal condition when subjected to heat stress.

### 3.3 The number of flowers increased under heat stress condition

The flower is an important generative organ for fruit formation. Statistical analysis showed a significant difference in the number of flowers among the tested genotypes under normal and heat-stress conditions. WT-MT had 94.5 and 122.5 more flowers in normal and heat-stress conditions, respectively. Meanwhile, *iaa9-3* and *iaa9-5* had 68.7 and 74.1 flowers under normal conditions and 91.6 and 84.8 flowers under heat-stress conditions, respectively. Raising the ambient temperature to 45°C during the growth period significantly increased the number of flowers on all tested plants. The number of flowers of WT-MT, *iaa9-3* and *iaa9-5* increased by 29.63%, 33.33% and 14.43%, respectively; under heat stress compared with those under normal condition (**Figure 3**).

### 3.4 Pollen viability decreased under heat-stress conditions

In the microscopic analysis of pollen viability, three groups, namely, viable, semi-viable and non-viable pollens were determined based on pollen viability level. Based on statistical data analysis, under normal conditions, the percentage of viable, semi-viable and non-viable pollens in the three genotypes of investigated plants did not show any significant difference. Among the three levels of the proportion of pollens, the group of viable pollens had the highest percentage compared with the semi-viable and non-viable pollen groups.

**Figure 4:**
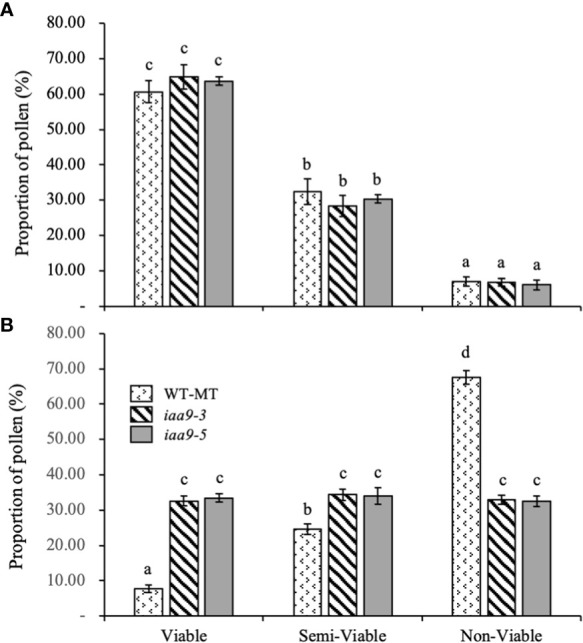
The proportion of pollen of WT-MT, *iaa9-3* and *iaa9-5* under two temperature conditions, i.e., **(A)** normal temperature and **(B)** heat stress condition. The average value ± standard error (SE) (n = 4) followed by the same lowercase letters is not significantly different based on Duncan’s Multiple Range Test at 5%.

This study proved that the decrease in pollen viability occurred under heat-stress conditions. Raising the temperature up to 45°C significantly reduced the level of pollen viability in all investigated genotypes. The decrease in the percentage of viable pollen under heat-stress conditions occurred in WT-MT, where the percentage of viable pollen decreased by almost 87.15% compared with that under normal condition. Mutations in the *IAA9* gene can prevent pollen damage under heat-stress condition, as indicated by the percentages of viable pollen in *iaa9-3* and *iaa9-5*, which reached 32.62% and 33.42%, respectively. The percentages decreased by 49.73% and 47.51%, respectively, compared with those under normal condition. The decrease in the number of viable pollens under heat-stress condition was caused by the increase in pollen sterility, which resulted in a very high number of non-viable pollens in WT-MT (67.57%) due to high temperature, and the decreases in *iaa9-3* and *iaa9-5* reached 32.97% and 32.56%, respectively (**Figure 4B**).

### 3.5 iaa9-3 and iaa9-5 can produce fruit under heat stress

**Figure 5:**
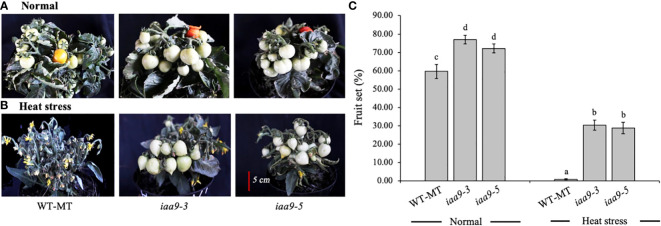
Fruit set of WT-MT, *iaa9-3* and *iaa9-5* under two temperature conditions, i.e., **(A) **normal temperature and **(B)** heat stress condition. **(C)** The average value of fruit set under normal temperature and heat stress conditions. The average value ± standard error (SE) (n = 4) followed by the same lowercase letters is not significantly different based on Duncan’s Multiple Range Test at 5%.

The fruit set is the percentage ratio of the number of fruits and the number of flowers in the same individual plant. Statistical analysis showed that the fruit set in *iaa9-3* and *iaa9-5* was significantly higher than that in WT-MT under normal and heat-stress conditions (**Figures 5A, B**, respectively). Plant capacity to produce fruit also differed between normal and heat-stress conditions, irrespective of genotypes. Under normal conditions, the fruit sets of *iaa9-3* and *iaa9-5* were 81.24% and 64.89% or 21.57% and 5.22% higher than that of WT-MT, respectively. The percentage of fruit set from all tested plants decreased dramatically under the heat-stress condition at 45°C (**Figure 5C**). Under the heat-stress condition, WT-MT was unable to produce fruits, whereas *iaa9-3* and *iaa9-5* did. Thus, mutations in the *IAA9* gene can increase the percentage of fruit set in *iaa9-3* and *iaa9-5* compared with WT-MT under heat-stress conditions. Although both mutants can produce fruit sets under heat-stress conditions, the fruit set was lower compared with that in normal conditions. The fruit sets of *iaa9-3* and *iaa9-5* under heat-stress conditions were 34.98% and 32.84% or 46.26% and 32.05% lower than those under normal conditions, respectively (**Figure 5C**).

### 3.6 iaa9-3 and iaa9-5 can produce normal fruit size under heat stress

**Figure 6:**
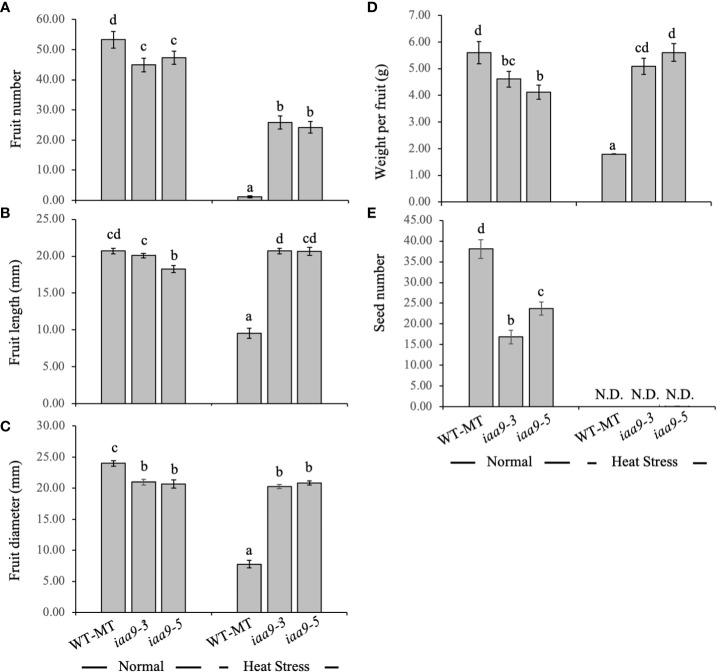
**(A)** Fruit number, **(B)** fruit length, **(C)** fruit diameter, **(D)** individual fruit weight, and **(E)** seed number of WT-MT, *iaa9-3* and *iaa9-5* under two temperature conditions, i.e., normal temperature and heat stress condition. The average value ± standard error (SE) (n = 4) followed by the same lowercase letters is not significantly different based on Duncan’s Multiple Range Test at 5%.

The number of fruits per plant, individual fruit weight and fruit diameter can be used as yield and yield quality variables of tomato fruit. Under the normal-temperature condition, all tested genotypes produced fruits. WT-MT plant produced the highest fruit number and significantly differed from the *iaa9-3* and *iaa9-5*. Under normal conditions, the number of fruits for WT-MT, *iaa9-3* and *iaa9-5* were 53.3, 47.8 and 46.8 fruits per plant, respectively. The heat condition affected the fruit yield of all investigated genotypes, with the lowest value detected in WT-MT. WT-MT had a small fruit size with no seed inside. However, *iaa9-3* and *iaa9-5* can still produce fruits under heat-stress condition, with the value being reduced compared with that under normal condition. Under heat condition, the reduction of fruit number varied compared to that in normal condition, i.e. 97.75%, 42.34% and 48.68% for WT-MT, *iaa9-3* and *iaa9-5*, respectively (**Figure 6A**).

Fruit size can be indicated by individual fruit weight, length and diameter. Under normal condition, *iaa9-3* and *iaa9-5* had a smaller fruit size, indicated by shorter fruit length and diameter and smaller fruit weight, compared with WT-MT. However, the fruit length of *iaa9-3* was comparable to that of WT-MT. Under the heat-stress condition, *iaa9-3* and *iaa9-5* resulted in a longer fruit length and higher fruit diameter compared with WT-MT (**Figures 6B–D**). Although *iaa9-3* and *iaa9-5* had a few fruit numbers under heat-stress condition, both mutants had a larger fruit size compared with that under normal condition. From the data obtained, **Figure 6** indicate a positive relationship between fruit number and fruit size, where more fruits were produced (**Figure 6A**), which resulted in smaller fruit sizes and weights (**Figures 6B–D**). Statistical data analysis showed that the individual fruit weights of *iaa9-3* and *iaa9-5* were significantly smaller than those of WT-MT under normal condition. However, both mutants had significantly higher fruit weight than WT-MT under heat-stress condition (**Figure 6B**).

In all investigated plants, the number of seeds was highly affected by genetic factors and environmental conditions. Mutation in the *IAA9* gene had a significant effect on the number of seeds. Under normal conditions, *iaa9-3* and *iaa9-5* had lower numbers of seeds compared with WT-MT, with reductions of up to 55.82% and 37.73%, respectively (**Figure 6E**). Heat stress significantly reduced the number of seeds in all investigated plants. Under the heat-stress condition, although *iaa9-3* and *iaa9-5* resulted in a larger fruit size compared with that under normal conditions, both mutants and WT-MT cannot produce seeds because of the heat-stress condition (**Figure 6E**).

### 3.7 Mutation in the *IAA9 gene* significantly reduced H_2_O_2_ content but showed increased antioxidant activity

**Figure 7:**
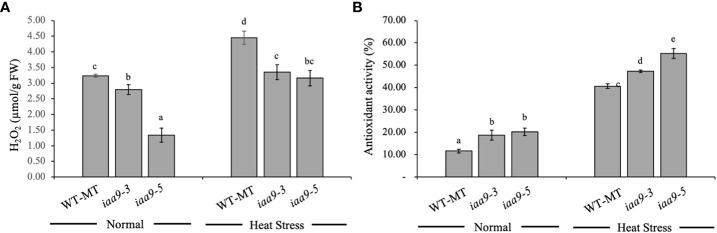
**(A)** H_2_O_2_ and **(B)** Antioxidant activity of WT-MT, *iaa9-3*, and *iaa9-5* leaves under two temperature conditions, normal temperature, and heat stress conditions. The average value ± standard error (SE) (n = 4) followed by the same lowercase letters is not significantly different based on Duncan’s Multiple Range Test at 5%.

H_2_O_2_ content was analysed to predict the accumulation of reactive oxygen species (ROS) within their leaves as an effect of *IAA9* mutation under two different environmental conditions. Statistical data analysis showed that H_2_O_2_ content differed under various plant genotypes and environmental conditions. Under normal conditions, the H_2_O_2_ content was lower than that under heat-stress conditions in all plant genotypes. Mutation in the *IAA9* gene significantly reduced H_2_O_2_ content in *iaa9-3* and *iaa9-5* compared with WT-MT, with values of 0.44 and 1.90 µmol/g FW, respectively, which is lower than that of WT-MT (**Figure 7A**). Environmental conditions significantly affected the content of H_2_O_2_ in plants. The rise in temperature resulted in the increased H_2_O_2_ content in all investigated plants, but *iaa9-3* and *iaa9-5* showed a lower H_2_O_2_ content compared with WT-MT. WT-MT had a significantly higher H_2_O_2_ content than *iaa9-3* and *iaa9-5*, but it was comparable between *iaa9-3* and *iaa9-5*. The increase in H_2_O_2_ content under heat-stress conditions varied among genotypes, with values of 1.22, 0.56 and 1.82 µmol/g FW for WT-MT, *iaa9-3* and *iaa9-5*, respectively. These values were higher than those under normal conditions (**Figure 7A**).

Antioxidant activity values directly influence ROS accumulation in plants. Antioxidant activity varied among genotypes under normal or heat-stress conditions. The statistical data analysis of antioxidant activity revealed a significant difference in antioxidant activity among genotypes under normal or heat-stress conditions (**Figure 7**). Under normal conditions, *iaa9-5* showed the highest leaf antioxidant activity, followed by *iaa9-3* whose value was significantly higher than that of WT-MT. A significant increase in antioxidant activity was detected when the plant was exposed to high temperatures or heat stress. Under heat-stress conditions, similar findings with normal conditions were observed. *iaa9-3* had the highest antioxidant activity, followed by *iaa9-3* and WT-MT, with increased antioxidant activities of 248.90%, 152.22% and 171.92% compared to the under normal conditions for WT-MT, *iaa9-3* and *iaa9-5*, respectively (**Figure 7C**).

## 4 Discussion

Tomato productivity is greatly affected by heat stress. Heat stress-induced male gametophyte abortion leads to fruit set reduction (Alsamir et al., 2021). The reduction of fruit set showed a consequence in reducing plant yield, which is a major problem in tomato production. The use of parthenocarpic tomatoes is an interesting approach to improving tomato production in heat-exposed locations, especially in the tropics. *iaa9-3* and *iaa9-5* resulted in a parthenocarpic tomato without any fertilisation process. Therefore, these tomatoes can be developed with a high-temperature characteristic for growing in the tropics. However, these mutants should be further evaluated to gain more information regarding their growth and development performances under heat-stress condition.

The diameter of the plant canopy is one of the plant growth variables that can show the response of plants to environmental conditions. Heat stress greatly affects plant growth, which can be characterised by a decline in vegetative growth, resulting in small plant size, such as the small canopy diameter observed compared with that under normal condition (**Figure 1**). Mutations in *iaa9-3* and *iaa9-5* resulted in smaller plant canopy diameters compared with that of WT-MT under normal or heat-stress conditions. The small number of lateral shoots produced caused the small diameter in the mutants. By contrast, the WT-MT compound leaf shape formed a longer leaf stalk and wider plant shape, resulting in a wider plant diameter (**Figure 1**). According to Valladares et al. (2007), the shape of a plant canopy is determined by the shape of leaves, lateral shoots and branching patterns. A high number of branching results in a wide plant canopy development.

The loss function of *IAA9* caused changes in the growth pattern of the shoot, which inhibited the growth of the lateral shoot. Inhibition of lateral shoot growth decreased the number of lateral shoots of *iaa9-3* and *iaa9-5* (**Figure 2**). Tezuka et al. (2011) stated that a high AUX level can inhibit lateral shoot regeneration in tomato plants. Wang et al. (2005) reported a contradictive study that the *AS-IAA9* plant showed decreased apical dominance. Heat stress also reduces plant growth by lowering plant stomatal conductance (Camejo et al., 2005). A low stomatal conductance reduces CO_2_ fixation while increasing leaf temperature, resulting in reduced photosynthesis and more oxidative damage in leaf tissue (Hemantaranjan, 2014).

The *iaa9-3* and *iaa9-5* experienced lateral shoot growth reduction, which led to a decline in the number of flowers compared with that in WT-MT under normal and heat-stress conditions (**Figures 2** and **3**). Under normal conditions, the flowering times of *iaa9-3* and *iaa9-5* were shorter than that of WT-MT. Possibly, mutations in *iaa9-3* and *iaa9-5* sped up flowering. According to Cheng and Zhao (2007) AUX plays an important role in the initiation of flower primordia. The shoot apical meristem turns into a flowering meristem that can initiate the floral meristem, in which AUX plays a role in regulating the proliferation of flower meristematic cells (Merelo et al., 2022). The role of AUX in primordial formation was evidenced by the increase in AUX concentration at the primordial tip of the flower (Vanneste and Friml, 2009). Accelerated flowering in WT-MT under heat stress was caused by the shortened plant life cycle (Solankey et al., 2015), but mutation in the *IAA9* gene can slow down the flowering process.

The highest number of lateral shoots was identified in WT-MT, resulting in a higher number of flowers on WT-MT either in normal or heat-stress conditions. Apical meristem can transform either into vegetative organs, such as leaves or reproductive organs, such as flowers (Ohta, 2018). Lateral shoots at certain points on determinate tomatoes can form bunches as a growth site of flowers and fruit (Park et al., 2016). AUX is directly involved in the formation of axillary buds. With the mutation of the *IAA9* gene, the number of lateral shoots in *iaa9-3* and *iaa9-5* mutants and the number of flowers produced decreased. Heat stress treatment up to 45°C caused a significant increase in the number of flowers on all tested genotypes than those under normal conditions, with a higher number of flowers observed on WT-MT than on *iaa9-3* and *iaa9-5* (**Figure 3**). The increased number of flowers under heat stress was in accordance with the statement of Park et al. (2016), who stated that high temperatures can induce flower growth as a plant adaptation to maintain its generation.

Pollen viability is a crucial factor in determining the success of fruit formation. The results of this study showed that mutations in *iaa9-3* and *iaa9-5* can increase the number of viable and semi-viable pollens and reduce the number of non-viable pollens under normal temperature or heat stress. According to Aloni et al. (2006) endogenous AUX plays an important role in the growth of the pollen tube that will penetrate the ovule, as evidenced by the increased concentration of IAA in the pistil after pollination. Sakata et al. (2010) stated that the application of AUX can increase the anther length, prevent pollen abortion and improve the viability of mature pollens. AUX can block transcriptional changes, which result in normal pollen production and prevent pollen sterility under heat stress (Higashitani, 2013). According to Pan et al. (2019) IAA can increase the fresh weight of stamens and pistils in tomato flowers when exposed to heat stress. At temperatures of 40°C–45°C, WT-MT had the lowest number of viable and semi-viable pollen and the highest number of non-viable pollens compared with both mutants. Thus, WT-MT had the highest potential for male sterility, which can affect fruit formation (**Figure 4**).

Tomato reproduction is extremely sensitive to heat stress. High temperatures can cause abortion of the male gametophyte, which leads to a reduction in fruit set. The increased fruit set in *iaa9-3* and *iaa9-5* under normal and heat-stress conditions was caused by the mutation in the *IAA9* gene. AUX synthesis can increase fruit set in tomato plants by preventing flowering and fruit fall (Batlang, 2008; Pramanik and P. Mohapatra, 2017). Mutations in the *IAA9* gene caused a reduction in the accumulation of mRNA, which triggered tomato fruit development before pollination, resulting in parthenocarpic fruit formation (Wang et al., 2005). AUX accumulation in the ovary vasculature and the micropylar pole of the ovule embryo sac for six days before anthesis was distributed to the ovule for two days before anthesis and finally localised to the interior and surface of the ovarian wall during anthesis (Pattison and Catalá, 2012). Under the heat-stress condition, WT-MT showed no ability to produce fruit (**Figure 5**) although it produced a large number of flowers (**Figure 4**). Solankey et al. (2015) stated that the rise in temperature affected the development of gametes and embryos and inhibited the ability of pollinated flowers to develop into seeds. Saito et al. (2011) stated that *iaa9-3* and *iaa9-5* are parthenocarpic tomato mutants with the ability to form fruit without fertilisation. Therefore, these tomatoes are very good candidates for tomato development in areas with the high-temperature condition that generally limits tomato productivity.

Both *iaa9-3* and *iaa9-5* can produce fruit without fertilisation due to a mutation in the *IAA9* gene under normal or heat-stress conditions. Heat stress caused a decrease in plant growth and development, which affected the yield, especially the number of fruits. Kumar et al. (2022) stated that exposure of plants to extreme temperatures limits their ability to produce fruit due to disruption of the pollination process. At normal temperatures, the number of fruits in both mutants was less than that in WT-MT. This finding was caused by the small number of flowers that formed in the mutants compared with WT-MT, which resulted in the formation of fewer fruits. Under the heat-stress condition, WT-MT did not produce fruits due to the failure of the fertilisation process as the effect of lowered pollen viability (**Figure 4**). Extremely high temperatures during the reproductive stage affect pollen viability, fertilisation and grain or fruit formation (Hatfield and Prueger, 2015). Non-viable pollens cannot pollinate flowers, causing failure in the fruit set (Sato et al., 2000). Meanwhile, *iaa9-3* and *iaa9-5* can produce fruits under heat stress. This result was due to the nature of the two tomatoes, which were parthenocarpic tomatoes, to form fruit without fertilisation (Ariizumi et al., 2013). The results showed that *iaa9-5* had the lowest fruit weight at normal temperatures. *IAA9* gene mutation in *iaa9-5* affected fruit weight due to genetic changes. Mutation locations in different alleles resulted in varied responses between *iaa9-3* and *iaa9-5*, such as fruit size and weight. Fruit weight was affected by the number of fruits on the plant. Under normal conditions, *iaa9-5* had a higher fruit number compared with that in heat-stress condition, but it had a lower fruit size (**Figure 6**). Burge et al. (1987) stated that fruit weight decreased with the increase in fruit number per vine in kiwifruit. A similar finding was reported by Puspitasari et al. (2014) who revealed that the more fruits produced, the less the individual fruit size.

*IAA9* gene mutations in *iaa9-3* and *iaa9-5* caused a decrease in the number of seeds under normal conditions. This study was in line with the research of Liu et al. (2018) on *SlARF5-*6 parthenocarpic transgenic tomato, which showed a high AUX response, resulting in a lower number of seeds compared with the WT. According to Takisawa et al. (2019) low seed production was attributed to two reasons, i.e. (1) formation of pseudo-embryo from the innermost layer of the embryo sac and (2) inhibition of pollen tube elongation. A few seeds that were found in both mutants at 40°C–45°C indicated that only a few viable pollens in both mutants can fertilise.

Heat stress increases ROS, such as H_2_O_2_, accumulation within plant tissues (Suzuki and Mittler, 2006). This continuous accumulation of ROS within plant tissues damages the cell plasma membrane (Dhanda and Munjal, 2009). In this study, *iaa9-3* and *iaa9-5* showed a lower H_2_O_2_ accumulation compared with WT-MT and higher antioxidant activities (**Figures 7A, B**). Antioxidants scavenge ROS and break it down into less radical compounds that are less harmful to plant cells (Moons, 2005). The higher antioxidant activity observed in *iaa9-3* and *iaa9-5* leaves was likely caused by the heightened AUX response in both *IAA9* mutants. Increased AUX concentration has been linked to increased antioxidant synthesis (Moons, 2005; Duan et al., 2010), DELLA protein stability (Paponov et al., 2008), and ethylene synthesis (Tognetti et al., 2012). AUX enhances antioxidant enzyme synthesis by activating the redox genes responsible for catalase, superoxide dismutase, guaiacol peroxidase, ascorbate peroxidase and glutathione-*S*-transferase synthesis (Tognetti et al., 2012; Sergiev et al., 2018).

In conclusion, the results showed that *iaa9-3* and *iaa9-5* exhibited a reduction in vegetative plant growth, delayed flowering time, increased number of flowers, ability to produce fruit and adaptability to heat-stress conditions. Thus, *iaa9-3* and *iaa9-5* can potentially contribute to breeding programs to generate new heat-tolerant commercial tomato cultivars.

## Data availability statement

The raw data supporting the conclusions of this article will be made available by the authors, without undue reservation.

## Author contributions

All authors listed have made a substantial, direct, and intellectual contribution to the work and approved it for publication.

## References

[B1] Aloni R. Aloni E. Langhans M. Ullrich C. I. (2006). Role of auxin in regulating arabidopsis flower development. Planta 223, 315–328. doi: 10.1007/s00425-005-0088-9 16208486

[B2] Alsamir M. Mahmood T. Trethowan R. Ahmad N. (2021). An overview of heat stress in tomato (Solanum lycopersicum l.). Saudi. J. Biol. Sci. 28, 1654–1663. doi: 10.1016/j.sjbs.2020.11.088 33732051 PMC7938145

[B3] Anas A. Wiguna G. Damayanti F. Mubarok S. Setyorini D. Ezura H. (2022). Effect of ethylene *Sletr1-2* receptor allele on flowering, fruit phenotype, yield, and shelf-life of four F1 generations of tropical tomatoes (*Solanum lycopersicum* l.). Horticulturae 8, 1098. doi: 10.3390/horticulturae8121098

[B4] Ariizumi T. Shinozaki Y. Ezura H. (2013). Genes that influence yield in tomato. Breed. Sci. 63, 3–13. doi: 10.1270/jsbbs.63.3 23641176 PMC3621442

[B5] Association of Official Analytical Chemists (1990). Ofdicial methods of analysis. (Gaithersburg, MD, USA: AOAC International).

[B6] Batlang U. (2008). Benzyladenine plus gibberellins (GA4+7) increase fruit size and yield in greenhouse-grown hot pepper (Capsicum annuum l.). J. Biol. Sci. 8, 659–662. doi: 10.3923/jbs.2008.659.662

[B7] Burge G. K. Spence C. B. Marshall R. R. (1987). Kiwifruit: Effects of thinning on fruit size, vegetative growth, and return bloom. New Z. J. Exp. Agric. 15, 317–324. doi: 10.1080/03015521.1987.10425577

[B8] Camejo D. Rodríguez P. Angeles Morales M. Miguel Dell’Amico J. Torrecillas A. Alarcón J. J. (2005). High temperature effects on photosynthetic activity of two tomato cultivars with different heat susceptibility. J. Plant Physiol. 162, 281–289. doi: 10.1016/j.jplph.2004.07.014 15832680

[B9] Cheng Y. Zhao Y. (2007). A role for auxin in flower development. J. Integr. Plant Biol. 49, 99–104. doi: 10.1111/j.1744-7909.2006.00412.x

[B10] Dhanda S. Munjal R. (2009). Cell membrane stability: Combining ability and gene effects under heat stress conditions. Cereal Res. Commun. 37, 409–417. doi: 10.1556/CRC.37.2009.3.10

[B11] Duan Q. Kita D. Li C. Cheung A. Y. Wu H.-M. (2010). FERONIA receptor-like kinase regulates RHO GTPase signaling of root hair development. Proc. Natl. Acad. Sci. 107, 17821–17826. doi: 10.1073/pnas.1005366107 20876100 PMC2955125

[B12] Ezura H. Hoshikawa K. Fukumoto S. Ooshima S. Mina A. (2019). Heat-tolerant tomato mutant and method for producing the same.

[B13] Guilfoyle T. J. Hagen G. (2007). Auxin response factors. Curr. Opin. Plant Biol. 10, 453–460. doi: 10.1016/j.pbi.2007.08.014 17900969

[B14] Hatfield J. L. Prueger J. H. (2015). Temperature extremes: Effect on plant growth and development. Weather. Clim. Extrem. 10, 4–10. doi: 10.1016/j.wace.2015.08.001

[B15] Hemantaranjan A. (2014). Heat stress responses and thermotolerance. Adv. Plants Agric. Res. 1, 1–10. doi: 10.15406/apar.2014.01.00012

[B16] Higashitani A. (2013). High temperature injury and auxin biosynthesis in microsporogenesis. Front. Plant Sci. 4. doi: 10.3389/fpls.2013.00047 PMC3593198 23483842

[B17] Iizuka K. Yonehara T. Itoh M. Kosugi Y. (2017). Estimating tree height and diameter at breast height (DBH) from digital surface models and orthophotos obtained with an unmanned aerial system for a Japanese cypress (Chamaecyparis obtusa) forest. Remote Sens. (Basel). 10, 13. doi: 10.3390/rs10010013

[B18] Kumar S. Thakur M. Mitra R. Basu S. Anand A. (2022). Sugar metabolism during pre- and post-fertilization events in plants under high temperature stress. Plant Cell Rep. 41, 655–673. doi: 10.1007/s00299-021-02795-1 34628530

[B19] Liu S. Zhang Y. Feng Q. Qin L. Pan C. Lamin-Samu A. T.. (2018). Tomato AUXIN RESPONSE FACTOR 5 regulates fruit set and development *via* the mediation of auxin and gibberellin signaling. Sci. Rep. 8, 2971. doi: 10.1038/s41598-018-21315-y 29445121 PMC5813154

[B20] Merelo P. González-Cuadra I. Ferrándiz C. (2022). A cellular analysis of meristem activity at the end of flowering points to cytokinin as a major regulator of proliferative arrest in arabidopsis. Curr. Biol. 32, 749–762.e3. doi: 10.1016/j.cub.2021.11.069 34963064

[B21] Moons A. (2005). Regulatory and functional interactions of plant growth regulators and plant glutathione s-transferases (GSTs). Vitamins & hormones 155–202. doi: 10.1016/S0083-6729(05)72005-7 16492471

[B22] Mubarok S. Ezura H. Qonit M. A. H. Prayudha E. Suwali N. Kurnia D. (2019b). Alteration of nutritional and antioxidant level of ethylene receptor tomato mutants, Sletr1-1 and Sletr1-2. Sci. Hortic. 256, 108546. doi: 10.1016/j.scienta.2019.108546

[B23] Mubarok S. Ezura H. Rostini N. Suminar E. Wiguna G. (2019a). Impacts of Sletr1-1 and Sletr1-2 mutations on the hybrid seed quality of tomatoes. J. Integr. Agric. 18 (5), 1170–1176. doi: 10.1016/S2095-3119(19)62614-6

[B24] Mubarok S. Hoshikawa K. Okabe Y. Yano R. Tri M. D. Ariizumi T.. (2019c). Evidence of the functional role of the ethylene receptor genes *SlETR4* and *SlETR5* in ethylene signal transduction in tomato. Mol. Genet. Genomics 294, 301–313. doi: 10.1007/s00438-018-1505-7 30382349

[B25] Mubarok S. Qonit M. A. H. Rahmat B. P. N. Budiarto R. Suminar E. Nuraini A. (2023). An overview of ethylene insensitive tomato mutants: Advantages and disadvantages for postharvest fruit shelf-life and future perspective. Front. Plant Sci. 14. doi: 10.3389/fpls.2023.1079052 PMC9911886 36778710

[B26] Oboulbiga E. B. Parkouda C. Sawadogo-Lingani H. Compaoré E. W. R. Sakira A. K. Traoré A. S. (2017). Nutritional composition, physical characteristics and sanitary quality of the tomato variety Mongol F1 from Burkina Faso. Food Nutr. Sci. 08, 444–455. doi: 10.4236/fns.2017.84030

[B27] Ohta K. (2018). “Branch formation and yield by flower bud or shoot removal in tomato,” in Physical methods for stimulation of plant and mushroom development (InTech). doi: 10.5772/intechopen.71519

[B28] Pan C. Yang D. Zhao X. Jiao C. Yan Y. Lamin-Samu A. T.. (2019). Tomato stigma exsertion induced by high temperature is associated with the jasmonate signalling pathway. Plant Cell Environ. 42, 1205–1221. doi: 10.1111/pce.13444 30203844

[B29] Paponov I. A. Paponov M. Teale W. Menges M. Chakrabortee S. Murray J. A. H.. (2008). Comprehensive transcriptome analysis of auxin responses in arabidopsis. Mol. Plant 1, 321–337. doi: 10.1093/mp/ssm021 19825543

[B30] Park H. J. Kim W.-Y. Pardo J. M. Yun D.-J. (2016). Molecular interactions between flowering time and abiotic stress pathways. 371–412. doi: 10.1016/bs.ircmb.2016.07.001 27692179

[B31] Pattison R. J. Catalá C. (2012). Evaluating auxin distribution in tomato (Solanum lycopersicum) through an analysis of the PIN and AUX/LAX gene families. Plant J. 70, 585–598. doi: 10.1111/j.1365-313X.2011.04895.x 22211518

[B32] Pham D. Hoshikawa K. Fujita S. Fukumoto S. Hirai T. Shinozaki Y.. (2020). A tomato heat-tolerant mutant shows improved pollen fertility and fruit-setting under long-term ambient high temperature. Environ. Exp. Bot. 178, 104150. doi: 10.1016/j.envexpbot.2020.104150

[B33] Pramanik K. P. Mohapatra,. P. (2017). Role of auxin on growth, yield and quality of tomato - a review. Int. J. Curr. Microbiol. Appl. Sci. 6, 1624–1636. doi: 10.20546/ijcmas.2017.611.195

[B34] Puspitasari Y. D. Aini N. Koesriharti K. (2014). Respon dua varietas tomat (Lycopersicon esculentum mill.) terhadap aplikasi zat pengatur tumbuh naphthalene acetic acid (Naa). Doctoral dissertation, Brawijaya University. Indonesia.

[B35] Rahmat B. P. N. Octavianis G. Budiarto R. Jadid N. Widiastuti A. Matra D. D.. (2023). SlIAA9 mutation maintains photosynthetic capabilities under heat-stress conditions. Plants 12, 378. doi: 10.3390/plants12020378 36679090 PMC9867002

[B36] Saito T. Ariizumi T. Okabe Y. Asamizu E. Hiwasa-Tanase K. Fukuda N.. (2011). TOMATOMA: A novel tomato mutant database distributing micro-tom mutant collections. Plant Cell Physiol. 52, 283–296. doi: 10.1093/pcp/pcr004 21258066 PMC3037083

[B37] Sakata T. Oshino T. Miura S. Tomabechi M. Tsunaga Y. Higashitani N.. (2010). Auxins reverse plant male sterility caused by high temperatures. Proc. Natl. Acad. Sci. 107, 8569–8574. doi: 10.1073/pnas.1000869107 20421476 PMC2889339

[B38] Sami F. J. Rahimah S. (2016). Uji aktivitas antioksidan ekstrak metanol bunga brokoli (*Brassica oleracea l. var. italica*) dengan metode DPPH (2,2 diphenyl-1-picrylhydrazyl) dan metode abts (2,2 azinobis (3-etilbenzotiazolin)-6-asam sulfonat). Jurnal. Fitofarmaka. Indonesia. 2, 107–110. doi: 10.33096/jffi.v2i2.179

[B39] Sato S. Peet M. M. Thomas J. F. (2000). Physiological factors limit fruit set of tomato (Lycopersicon esculentum mill.) under chronic, mild heat stress. Plant Cell Environ. 23, 719–726. doi: 10.1046/j.1365-3040.2000.00589.x

[B40] Sergiev I. Todorova D. Shopova E. Jankauskienė J. Jankovska-Bortkevič E. Jurkonienė S. (2018). Effects of auxin analogues and heat stress on garden pea. Zemdirbyste-Agriculture 105, 243–248. doi: 10.13080/z-a.2018.105.031

[B41] Solankey S. S. Singh R. K. Baranwal D. K. Singh D. K. (2015). Genetic expression of tomato for heat and drought stress tolerance: An overview. Int. J. Vegetable. Sci. 21, 496–515. doi: 10.1080/19315260.2014.902414

[B42] Suminar E. Budiarto R. Nuraini A. Mubarok S. Ezura H. (2022). Morpho-physiological responses of iaa9 tomato mutants to different levels of PEG simulated drought stress. Biodivers. J. Biol. Diversity 23 (6), 3115–3126. doi: 10.13057/biodiv/d230639

[B43] Suzuki N. Mittler R. (2006). Reactive oxygen species and temperature stresses: A delicate balance between signaling and destruction. Physiol. Plant 126, 45–51. doi: 10.1111/j.0031-9317.2005.00582.x

[B44] Takisawa R. Koeda S. Nakazaki T. (2019). Effects of the &lt;i<pat-2&lt;/i< gene and auxin biosynthesis inhibitor on seed production in parthenocarpic tomatoes (&lt;i<Solanum lycopersicum&lt;/i< l.). Hort. J. 88, 481–487. doi: 10.2503/hortj.UTD-085

[B45] Tezuka T. Harada M. Johkan M. Yamasaki S. Tanaka H. Oda M. (2011). Effects of auxin and cytokinin on *In vivo* adventitious shoot regeneration from decapitated tomato plants. HortScience 46, 1661–1665. doi: 10.21273/HORTSCI.46.12.1661

[B46] Tognetti V. B. Mühlenbock P. van Breusegem F. (2012). Stress homeostasis - the redox and auxin perspective. Plant Cell Environ. 35, 321–333. doi: 10.1111/j.1365-3040.2011.02324.x 21443606

[B47] Valladares F. Gianoli E. Gómez J. M. (2007). Ecological limits to plant phenotypic plasticity. New Phytol. 176, 749–763. doi: 10.1111/j.1469-8137.2007.02275.x 17997761

[B48] Vanneste S. Friml J. (2009). Auxin: A trigger for change in plant development. Cell 136, 1005–1016. doi: 10.1016/j.cell.2009.03.001 19303845

[B49] Wang H. Jones B. Li Z. Frasse P. Delalande C. Regad F.. (2005). The tomato *Aux*/*IAA* transcription factor *IAA9* is involved in fruit development and leaf morphogenesis. Plant Cell 17, 2676–2692. doi: 10.1105/tpc.105.033415 16126837 PMC1242265

